# Design of a Human Defensin 5-Derived Linear Peptide with Potent Antibacterial Efficacy Against Carbapenem-Resistant *Acinetobacter baumannii*

**DOI:** 10.3390/molecules31213885

**Published:** 2026-10-28

**Authors:** Gaomei Zhao, Chengzhang Tan, Xin Li, Chenwenya Li, Mingrui Shu, Chao Wang, Kainuo Quan, Yongwu He, Jining Gao, Songling Han, Junping Wang, Cheng Wang

**Affiliations:** 1 State Key Laboratory of Trauma and Chemical Poisoning, Institute of Combined Injury of PLA, College of Preventive Medicine, Army Medical University, Chongqing 400038, China; zhaogaomei@tmmu.edu.cn (G.Z.);; 2 College of Basic Medicine, Army Medical University, Chongqing 400038, China

**Keywords:** carbapenem-resistant *Acinetobacter baumannii*, human defensin, arginine, antibacterial action, biocompatibility

## Abstract

The increasing incidence of infections caused by carbapenem-resistant *Acinetobacter baumannii* (CRAB) poses a serious threat to global public health, yet treatment options remain scarce. Human defensins (HDs) represent a promising alternative to conventional antibiotics for combating superbugs; however, their structural complexity often hinders synthetic accessibility. Here, we report the rational design of HD5lp5, a linear peptide derived from HD5 through the removal of disulfide bonds and the introduction of arginine residues. This derivative exhibits potent bactericidal activity against CRAB and retains bioactivity in complex physiological environments. Mechanistically, HD5lp5 binds to bacterial lipid A with a dissociation constant of 1.81 nM and destroys membrane integrity in a dose-dependent manner, inducing the leakage of intracellular contents. Beyond membrane disruption, its structural flexibility enables translocation into bacteria. In vitro translation assay reveals that HD5lp5 inhibits ribosomal translation with a half-maximal inhibitory concentration of 0.38 μM. Notably, HD5lp5 exhibits no apparent toxicity in vivo, and it effectively eliminates CRAB colonisation in murine models of intra-abdominal sepsis and irradiation-assisted pulmonary infection, thereby increasing survival rates. Our findings establish HD5lp5 as a promising preclinical candidate for treating life-threatening CRAB.

## 1. Introduction

Since the discovery of penicillin in 1928, which initiated the golden age of natural-product antibiotic discovery, clinicians have established extensive drug reserves to combat various bacteria. However, the advantages of traditional antibiotics are gradually disappearing in the 21st century, a decline driven by widespread drug abuse and a lag in antibiotic development relative to the rapid evolution of antimicrobial resistance (AMR) [1]. According to recent global estimates, AMR contributed to 4.71 million deaths in 2021, with resistant bacteria such as *Acinetobacter baumannii* (*A. baumannii*) and *Staphylococcus aureus* accounting for 1.14 million of these fatalities [2]. *A. baumannii* is a Gram-negative opportunistic bacterium that survives harsh conditions and readily acquires multidrug resistance. Most alarmingly, carbapenem-resistant *A. baumannii* (CRAB), a critical healthcare-associated pathogen in intensive care units (ICUs) that causes severe infections with limited treatments and high mortality, has spread globally [3]. Considering this escalating resistance crisis, the World Health Organization (WHO) placed CRAB in its highest-risk critical priority category on its latest Bacterial Priority Pathogens List [4].

To address the shortage of effective therapeutics against CRAB-induced infections, alternative strategies, such as drug repurposing, phage therapy, and antimicrobial peptide (AMP) treatments, are needed [5]. AMPs are amphipathic molecules that are widely distributed across all kingdoms of life and have evolved multiple anti-infective functions during evolutionary confrontation with microorganisms [6]. Among them, peptides with low immunogenicity and slow emergence of AMR, such as human defensins (HDs), are highly promising candidates [7]. HDs constitute a family of endogenous human AMPs and are characterised by low molecular weight, cysteine (Cys)-rich sequences, and high numbers of positively charged amino acids [8]. On the basis of the connectivity of their intramolecular disulfide pairings, HDs are divided into two subfamilies, α and β defensins [9]. Both α-defensins and β-defensins adopt a characteristic three-stranded β-sheet fold stabilised by three disulfide bonds, while a small helical region is also present in some β-defensins [8]. Among human α defensins, human defensin 5 (HD5), an AMP secreted by intestinal Paneth cells, has high broad-spectrum antibacterial activity [10], thus providing an inspiring template for the design of antibiotics for the treatment of CRAB.

Despite its potent antibacterial properties, the therapeutic translation of HD5 is hindered by the structural complexity conferred by its three intramolecular disulfide bonds, which substantially increase production costs and manufacturing difficulty. Disulfide bond removal offers a straightforward strategy to simplify the peptide architecture. However, linear peptides are generally susceptible to rapid proteolytic degradation and renal filtration, resulting in a short half-life in vivo [11]. To overcome this limitation, enhancing the rapid bactericidal activity of linear HD5 represents a rational strategy. Indeed, most clinically approved peptide drugs are characterised by rapid clearance and rapid onset of action [12]. Arginine (Arg), a naturally occurring cationic residue enriched in HDs, possesses a bidentate binding capability towards lipid headgroups and glycosaminoglycans [13,14]. Given that Arg facilitates the rapid attachment of AMPs to microbial membranes [15], the strategic incorporation of Arg into linear HD5 constitutes a promising approach to potentiate its rapid bactericidal efficacy.

In this study, we developed an Arg-aided structural simplification strategy to convert HD5 into a peptide antibiotic candidate. A series of HD5-derived linear peptides (HD5lps) was generated by removing the disulfide bonds and incorporating Arg residues. Among these peptides, HD5lp5 was identified as a lead candidate exhibiting potent and rapid bactericidal activity against CRAB. The underlying antibacterial mechanism was investigated using biolayer interferometry (BLI) and translation assays. Following toxicological evaluation, the therapeutic efficacy of HD5lp5 was assessed in murine models of intra-abdominal sepsis and irradiation-assisted local pulmonary infection. Our findings show that HD5lp5 is a fast-acting linear peptide with potent antibacterial activity against CRAB.

## 2. Results and Discussion

### 2.1. Introduction of Arg Enhances the Antibacterial Activity of Linear HD5

**Figure 1:**
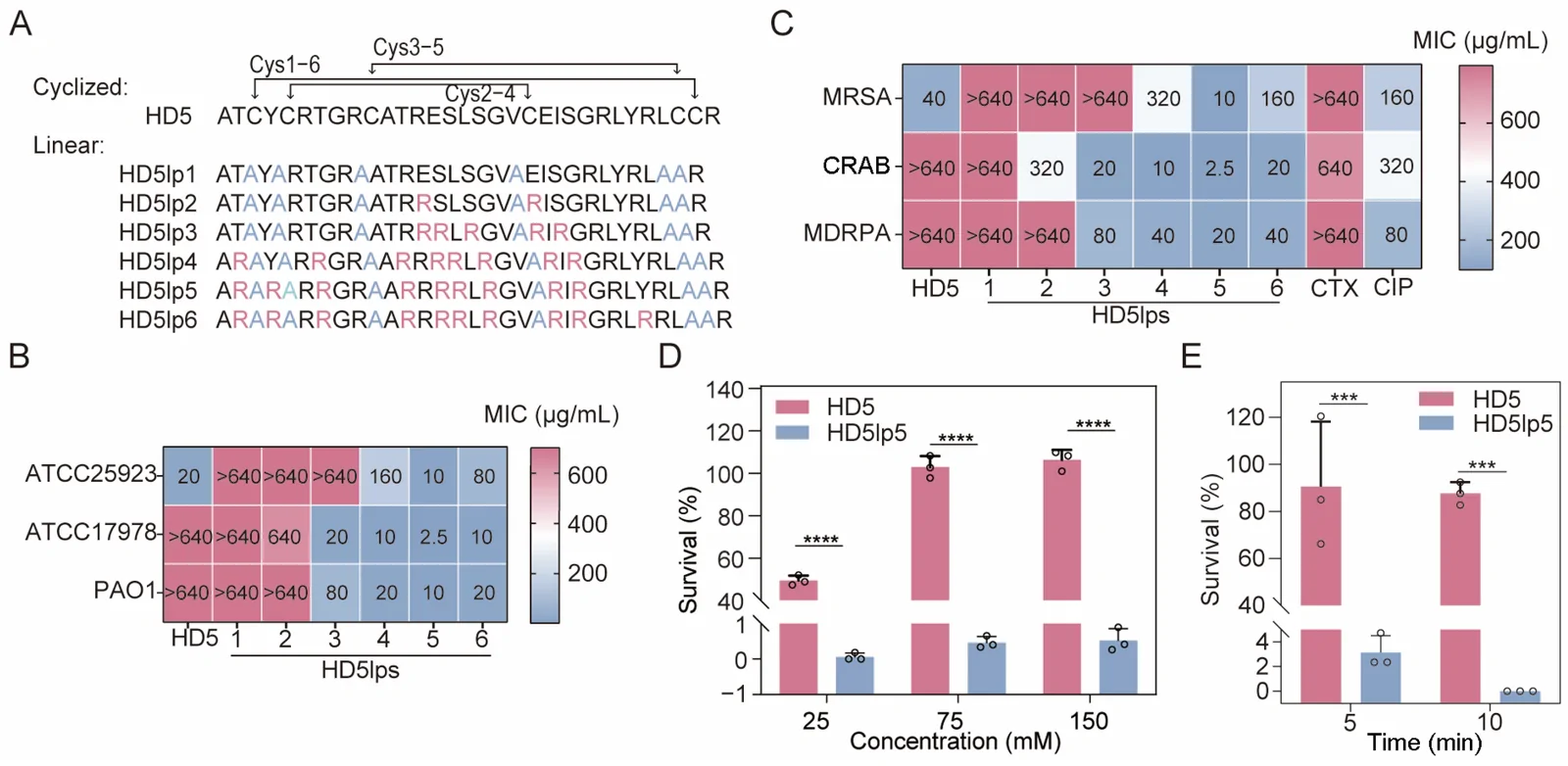
Antibacterial assessment of human defensin 5 (HD5) and HD5-derived linear peptides (HD5lps). (**A**) Amino acid sequences of HD5 and HD5lps. (**B**) Heatmap depicting the differences in the minimal inhibitory concentrations (MICs) of HD5 and HD5lps for clearing *A. baumannii* (ATCC 17978), *P. aeruginosa* (PA01), and *S. aureus* (ATCC 25923). (**C**) Heatmap depicting the differences in the MICs of HD5 and HD5lps for clearing carbapenem-resistant *Acinetobacter baumannii* (CRAB), multidrug resistant *P. aeruginosa* (MDRPA), and methicillin-resistant *S. aureus* (MRSA). CTX, cefotaxime; CIP, ciprofloxacin. (**D**) Salt-resistant antibacterial action of HD5 and HD5lp5 against CRAB. The results are shown as the mean ± standard deviation (SD) of three biological replicates (*n* = 3), with technical replicates averaged prior to analysis. ****, *p* < 0.0001, as determined by two-way analysis of variance (ANOVA) followed by simple effects analysis using Sidak’s multiple comparisons test. (**E**) Antibacterial activity of HD5 and HD5lp5 in the presence of 20% mouse serum. The results are shown as the mean ± SD of three biological replicates (*n* = 3), with technical replicates averaged prior to analysis. ***, *p* < 0.001, as determined by two-way ANOVA followed by simple effects analysis using Sidak’s multiple comparisons test.

To simplify the HD5 structure, we replaced its Cys residues with alanine (Ala) to generate the linear peptide HD5lp1 (Figure 1A). Given that nonpolar amino acids contribute to the membrane penetration of AMPs via hydrophobic interactions [16], the polar residues in HD5lp1, including glutamate (Glu), serine (Ser), threonine (Thr), and tyrosine (Tyr), were subsequently replaced with Arg to produce the derivatives HD5lp2–6 (Supplementary Table S1). Three standard bacterial strains, namely, Gram-negative *A. baumannii* (ATCC 17978) and *Pseudomonas aeruginosa* (*P. aeruginosa*, PAO1) and Gram-positive *S. aureus* (ATCC 25923), were used for antibacterial evaluation. In terms of the minimal inhibitory concentrations (MICs) determined by the broth microdilution method, compared with HD5, HD5lp1 was less effective at eliminating *S. aureus* (Figure 1B). This might be because the Cys residues in native HD5 form three specific intramolecular disulfide bonds (Cys1–Cys6, Cys2–Cys4, and Cys3–Cys5), which are required for its bactericidal activity, particularly against Gram-positive bacteria [17]. Notably, the introduction of Arg residues improved the antibacterial activity of HD5lp1 to varying degrees, and among the tested variants, HD5lp5, in which nine residues (Thr^2^, Tyr^4^, Thr^7^, Thr^12^, Glu^14^, Ser^15^, Ser^17^, Glu^21^, and Ser^23^) were replaced with Arg, exhibited the best performance. Compared with native HD5, HD5lp5 showed significantly enhanced bactericidal activity against *A. baumannii* and *P. aeruginosa*, with MICs reduced by at least 256-fold (from >640 µg/mL to 2.5 µg/mL) and 64-fold (from >640 µg/mL to 10 µg/mL), respectively (Figure 1B). Additionally, a twofold reduction in the MIC against *S. aureus* was also observed for HD5lp5.

Although membrane attraction driven by electrostatic interactions is the initial step for most cationic AMPs [18], our recent study revealed that an increase in net charge does not invariably lead to enhanced antibacterial activity [19]. The observation that HD5lp6 exhibits weaker antibacterial activity than HD5lp5 does provides further support for this conclusion. HD5lp6 differs from HD5lp5 only in the substitution of Tyr^27^ with Arg. Because Tyr^27^ is located within the functional motif (R^25^L^26^Y^27^R^28^) of HD5, which is critical for bacterial clearance [20], we speculate that this active domain may also play an indispensable role in mediating the bactericidal activity of linear HD5.

### 2.2. HD5lp5 Exhibits Potent Antibacterial Activity Against CRAB

To evaluate the activity of HD5lps against resistant bacteria, three additional strains, namely, Gram-negative CRAB (clinical strain *No. 1* [19]; resistant to: doxycycline, piperacillin/tazobactam, cotrimoxazole, levofloxacin, CIP, tobramycin, meropenem, imipenem, cefepime, ceftazidime, and cefoperazone/sulbactam; susceptible to: minocycline, tigecycline, and colistin), multidrug resistant *P. aeruginosa* (MDRPA) [21], and Gram-positive MRSA, were tested. The conventional antibiotics ciprofloxacin (CIP) and cefotaxime (CTX) were used as references. Consistent with the results obtained for standard strains, compared with HD5, HD5lp5 demonstrated superior efficacy in clearing resistant bacteria (Figure 1C). Remarkably, the MICs of HD5lp5 against CRAB, MDRPA, and MRSA were 256-fold (from 320 µg/mL to 2.5 µg/mL), 4-fold (from 80 µg/mL to 20 µg/mL), and 16-fold (from 160 µg/mL to 10 µg/mL) lower, respectively, than those of CIP. Moreover, CTX weakly inhibited these resistant strains (MICs ≥ 640 µg/mL). Because the shielding effect induced by high concentrations of salt ions in vivo attenuates the activity of AMPs [22,23], we next evaluated the antibacterial activity of HD5lp5 against CRAB in saline. As depicted in Figure 1D, 10 µg/mL HD5 had a minimal effect on CRAB survival at NaCl concentrations above 75 mM, whereas the same concentration of HD5lp5 resulted in greater than 99% clearance at 150 mM NaCl. Moreover, HD5lp5 completely eliminated CRAB at 25 µg/mL in 20% mouse serum within 10 min, whereas HD5 remained completely ineffective under the same conditions (Figure 1E). These data show that HD5lp5 can overcome the shielding effects of salt ions and exhibit rapid and robust antibacterial activity against CRAB.

### 2.3. HD5lp5 Outperforms HD5 in Binding to and Penetrating Bacterial Membranes

**Figure 2:**
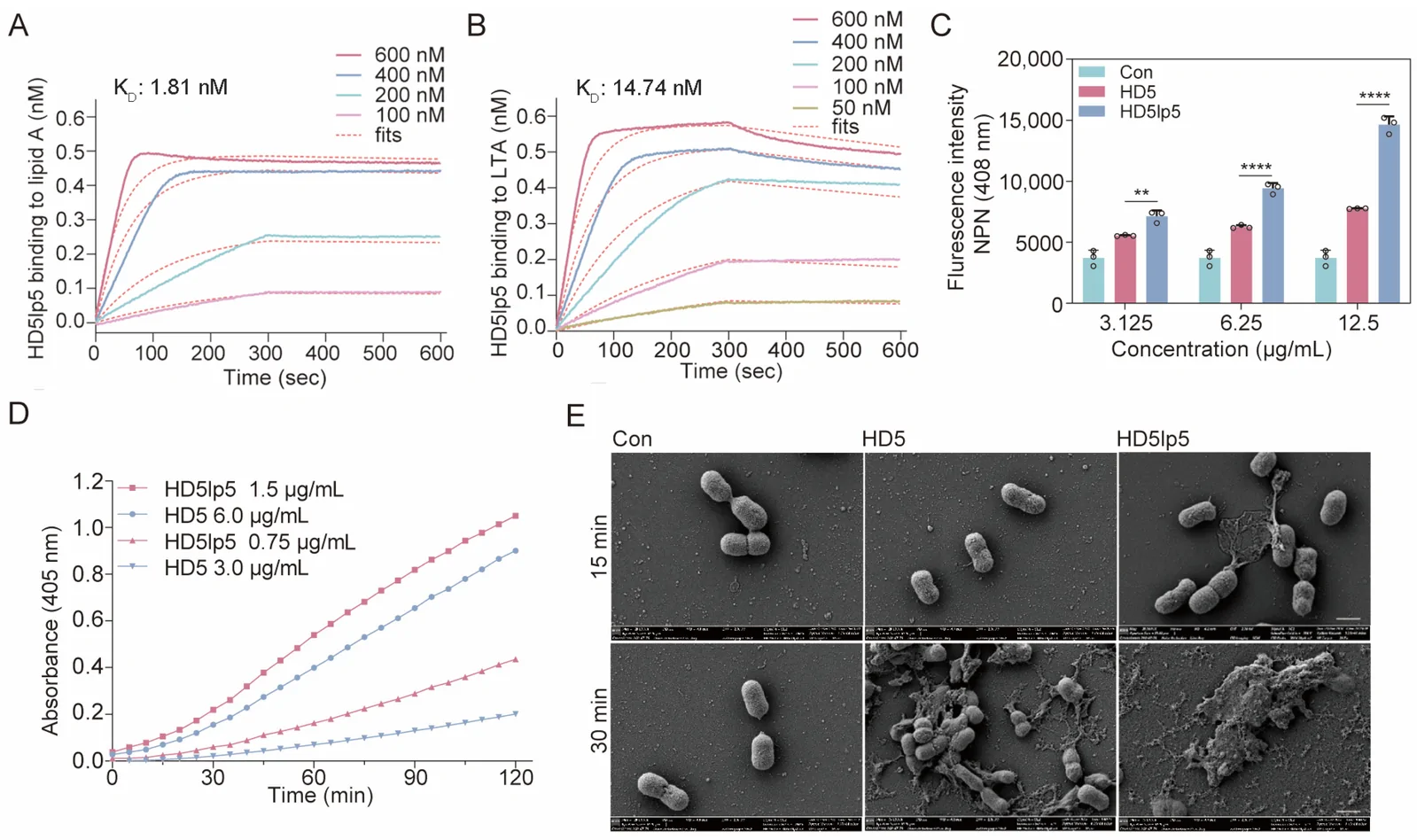
Membrane attraction and penetration of HD5lp5. (**A**) Binding kinetics of HD5lp5 to bacterial lipid A and (**B**) lipoteichoic acid (LTA). (**C**) Fluorescence intensity of 1-N-phenyl naphthylamine (NPN) equilibrated with CRAB in the presence of HD5 and HD5lp5. The emission wavelength of NPN was fixed at 350 nm. The results are shown as the mean ± SD of three biological replicates (*n* = 3), with technical replicates averaged prior to analysis. **, *p <* 0.01; ****, *p* < 0.0001, as determined by two-way ANOVA followed by simple effects analysis using Sidak’s multiple comparisons test. (**D**) Bacterial inner membrane permeabilization induced by HD5 and HD5lp5. (**E**) Scanning electron microscopy (SEM) images showing the morphological changes in CRAB cells exposed to HD5 and HD5lp5. Scale indicates 1 μm.

The antibacterial action of AMPs depends on their ability to first localise to the bacterial surface and subsequently penetrate the membrane [7,24]. To elucidate the improved antibacterial effect of HD5lp5, we used high-throughput biolayer interferometry (BLI) to determine its binding affinity to key components of the bacterial membrane. HD5lp5 bound to lipid A (Figure 2A) and lipoteichoic acid (LTA, Figure 2B) with dissociation constants (K_D_) of 1.81 nM and 14.74 nM, respectively, which are 66.3- and 14.9-fold lower than those of natural HD5 [20]. To further assess outer membrane penetration, we performed a 1-N-phenyl naphthylamine (NPN) uptake assay. Compared with treatment with equivalent concentrations of HD5, treatment with 3.125, 6.25, and 12.5 µg/mL HD5lp5 resulted in a significantly greater fluorescence intensity in CRAB (Figure 2C). Consistent with these findings, an inner membrane permeabilization assay, which indirectly reflects AMP-induced membrane damage in *A. baumannii* [19], revealed that the production of *ο*-nitrophenol (ONP), derived from the hydrolysis of 2-nitrophenyl β-D-galactopyranoside (ONPG) by cytoplasmic β-galactosidase [17], also increased to a greater extent in bacteria treated with HD5lp5 than in HD5-treated cells (Figure 2D). Scanning electron microscopy (SEM) further revealed that after coincubation with 10 µg/mL of each peptide at 37 °C for 15 or 30 min, compared with HD5, HD5lp5 induced more severe membrane disruption and more pronounced leakage of intracellular contents in CRAB (Figure 2E). Considering that Arg residues help AMPs associate with membrane components [25] and can induce negative Gaussian curvature to facilitate pore formation on bacterial membranes [26], the introduction of Arg residues may account for the enhanced lipid A binding of HD5lp5 and its greater membrane disruption relative to that of HD5.

### 2.4. HD5lp5 Inhibits Translation In Vitro

**Figure 3:**
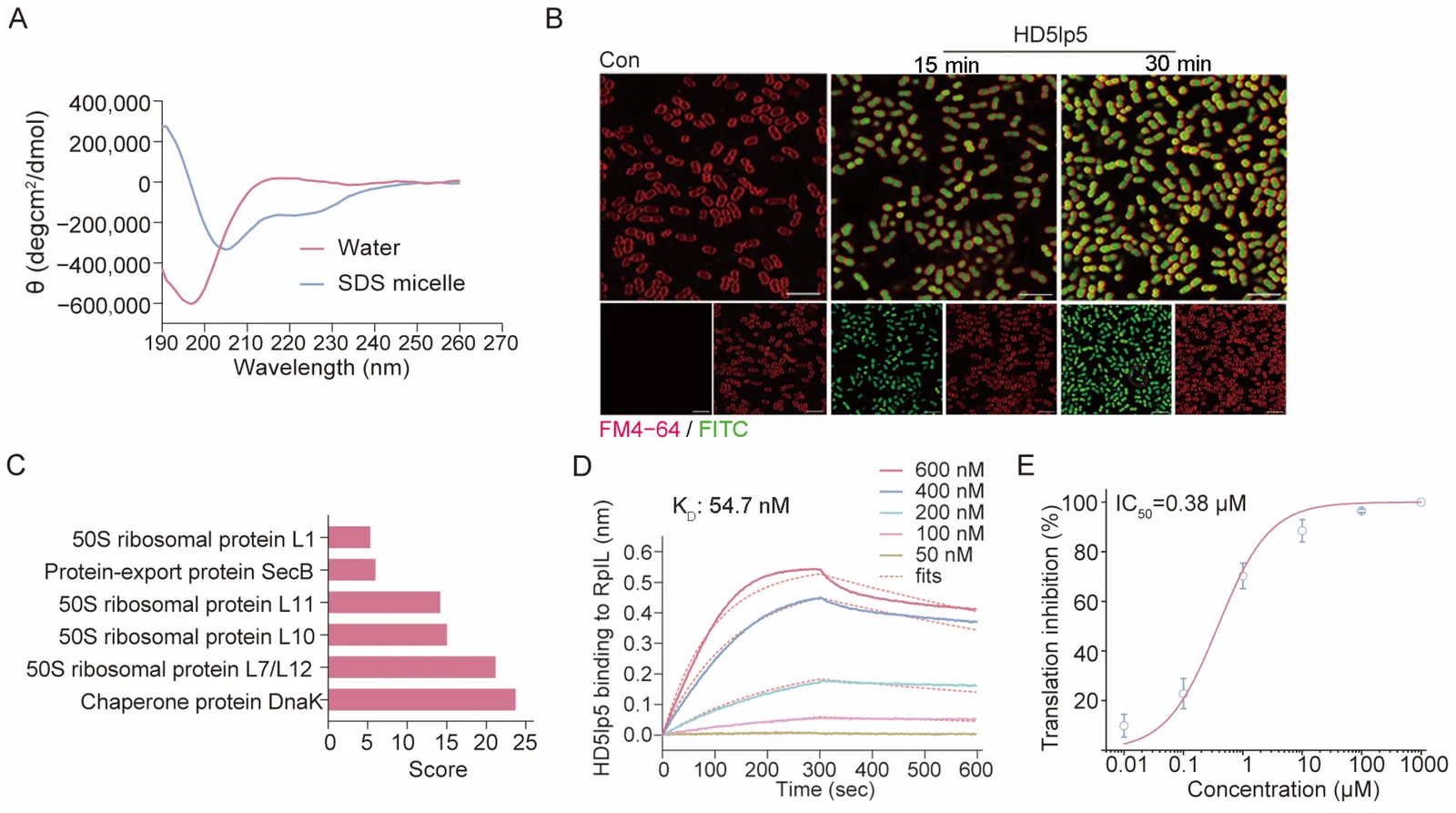
Ribosomal targeting and inhibition of HD5lp5. (**A**) Circular dichroism (CD) spectra revealing the conformation of HD5lp5 in water and sodium dodecyl sulfate (SDS) micelles. (**B**) Confocal microscopy images showing the molecular location of HD5lp5 in CRAB. HD5lp5 was labelled with FITC (488/525 nm, green fluorescence), and the bacterial membrane was stained with FM4–64 (565/744 nm, red fluorescence). Scale indicates 5 μm. (**C**) Identification of candidate proteins that interact with HD5lp5. The histogram shows the mass spectrometry (MS)-based scores of the six enriched proteins specifically pulled down by biotinylated HD5lp5. (**D**) Binding kinetics of HD5lp5 to recombinant L7/L12 protein. (**E**) In vitro translation assay demonstrating the inhibition of protein synthesis by HD5lp5. The data are presented as the mean ± SD of triplicate experiments.

The structural features of peptides determine their functions. Constrained by three pairs of intramolecular disulfide bonds, native HD5 adopts a rigid β-sheet-stranded structure that limits its ability to translocate into the bacterial cytoplasm [22]. In contrast, circular dichroism (CD) spectroscopy revealed that HD5lp5 adopts a random coil conformation in aqueous solution but transitions to an α-helical structure in sodium dodecyl sulfate (SDS) lipid micelles, as manifested by a positive peak at wavelengths between 190 and 193 nm and double minima at wavelengths of 205 and 220 nm (Figure 3A), indicating the structural flexibility of this peptide. This property enables HD5lp5 to translocate into bacteria (Figure 3B), suggesting the presence of intracellular targets for the peptide. To test this hypothesis, we synthesised biotin-labelled HD5lp5 and incubated it with *A. baumannii* (ATCC 17978) for 2 h. Subsequently, proteins were extracted and enriched using streptavidin magnetic beads. Mass spectrometry (MS) analysis indicated six potential target proteins (Figure 3C, Supplementary Table S2). Among these, the ribosomal proteins L7/L12 (encoded by the *RplL* gene), L10, L11, and L1 are components of the large 50S ribosomal subunit. BLI assays revealed that HD5lp5 bound to recombinant L7/L12 protein (Supplementary Figure S1), with a K_D_ of 54.7 nM (Figure 3D), suggesting that HD5lp5 can directly target the bacterial ribosome. Furthermore, an in vitro translation assay revealed that HD5lp5 inhibited bacterial translation in a dose-dependent manner, with a half-maximal inhibitory concentration (IC_50_) of 0.38 μM (Figure 3E).

Ribosomes translate genetic information into proteins and play an essential role in all cells. The bacterial ribosome, one of the primary antibiotic targets, is composed of a large 50S subunit and a small 30S subunit containing three ribosomal RNAs (16S, 23S and 5S) and approximately 54 ribosomal proteins [27]. Inhibitors targeting either the 50S or the 30S ribosomal subunit are successful bactericides and constitute more than half of clinical antibiotics [28]. Similar to lincosamides, streptogramins and phenicols [29], our results revealed that HD5lp5 associates with the 50S subunit, particularly the L7/L12, L11, and L10 proteins, which are core components of the ribosome L7/L12 stalk [30]. As one of the two protein-rich regions on the 50S subunit, the L7/L12 stalk is a highly mobile lateral protuberance structurally and acts as a ribosomal factor-binding region and a GTPase-associated centre [31]. Furthermore, the L7/L12 stalk region provides the main sites for aminoacyl-tRNA synthetase association and mediates the recycling of transfer RNAs, allowing efficient ribosomal protein synthesis [32]. Previous studies have shown that thiostrepton, a member of the thiazole class of antibiotics, can inhibit bacterial protein synthesis by binding to this stalk [33]. The association of HD5lp5 with the ribosomal protein L7/L12, together with its inhibitory effect on ribosomal translation, suggests a potential ribosomal-targeting mechanism underlying its bactericidal activity against CRAB.

### 2.5. HD5lp5 Exhibits No Apparent Toxicity In Vitro and In Vivo

**Figure 4:**
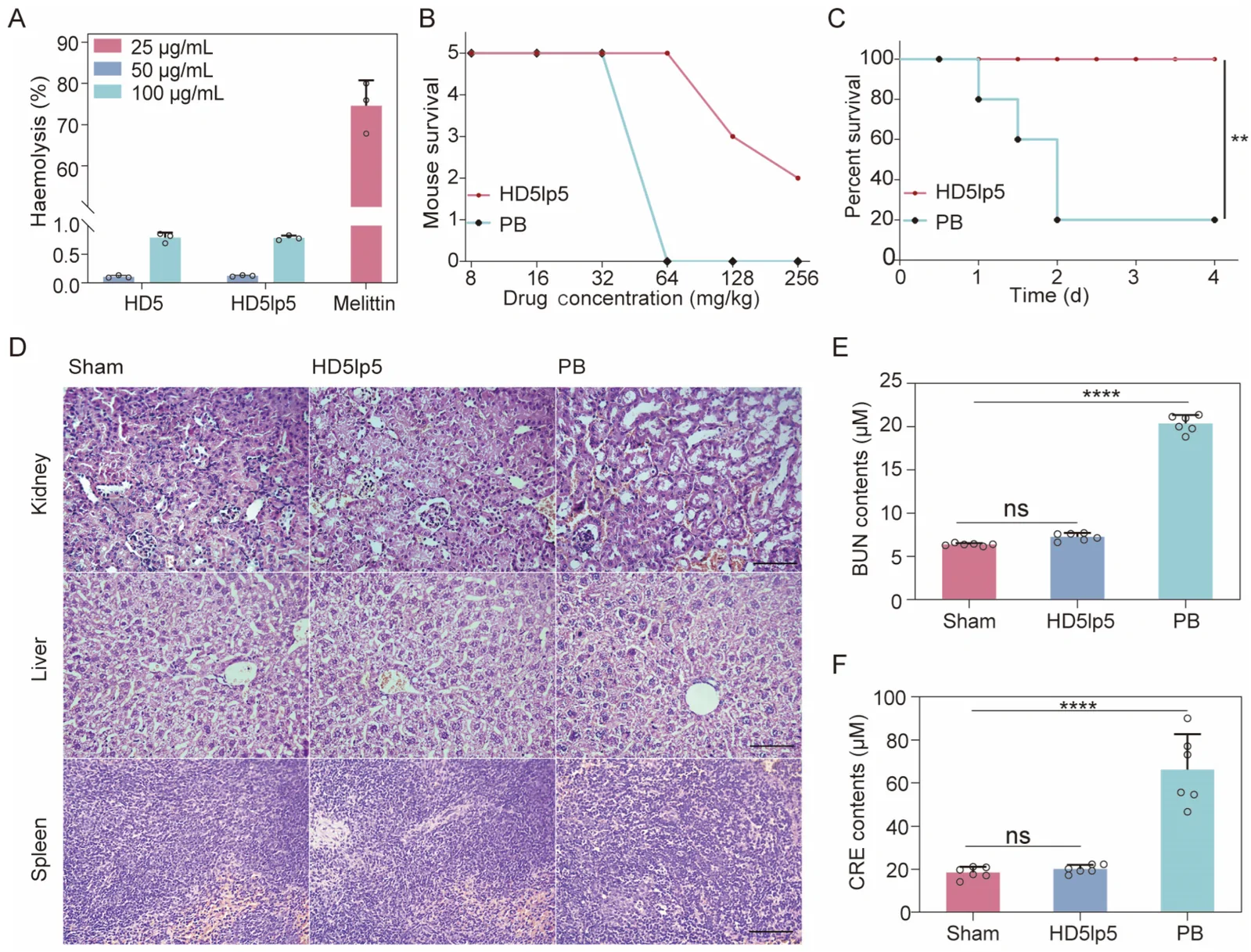
Toxicological evaluation of HD5lp5. (**A**) Haemolysis induced by different doses of HD5 and HD5lp5. Melittin (25 µg/mL) was used as a positive control. The results are shown as the mean ± SD of three biological replicates (*n* = 3), with technical replicates averaged. (**B**) Survival of mice that received different doses of HD5lp5 and polymyxin B (PB). Each group included five mice. (**C**) Percent survival of mice treated with HD5lp5 and PB. Each group included ten mice. Survival curves were plotted using the Kaplan–Meier method. **, *p* < 0.01, as determined by the log-rank test. (**D**) Hematoxylin and eosin (HE) staining of mouse kidney, liver, and spleen tissues. Scale indicates 200 μm. (**E**) Contents of blood urea nitrogen (BUN) and (**F**) creatinine (CRE) in mouse blood. The results are shown as the mean ± SD of six biological replicates (*n* = 6). ns, not significant; ****, *p <* 0.0001, as determined by one-way ANOVA followed by Tukey’s multiple comparisons test.

Given that cytotoxicity hinders the further development of many potent AMPs, such as latarcin and melittin [34,35], as an antibiotic candidate, we next carried out in vitro and in vivo experiments to evaluate the toxicity of HD5lp5. Less than 1% haemolysis was observed for HD5lp5 at 100 μg/mL (Figure 4A), which was much lower than that induced by 25 μg/mL melittin. No significant difference was observed between HD5 and HD5lp5. Additionally, the peptide was nontoxic to human umbilical vein endothelial cells (HUVECs) (Supplementary Figure S2) at concentrations up to 100 μg/mL. For in vivo assessment, increasing doses of HD5lp5 were administered by intraperitoneal injection to BALB/c mice. Polymyxin B (PB), a polymyxin antibiotic used in clinical practice to treat infections caused by drug-resistant Gram-negative bacteria, was used as a control. After single-dose drug administration, mouse survival was monitored for 72 h. At a dose of 64 mg/kg, HD5lp5 did not affect the survival rate of the mice, whereas PB induced complete lethality under the same conditions (Figure 4B). Moreover, the mice that received 64 mg/kg HD5lp5 twice daily survived after 4 days (Figure 4C), whereas 80% of the mice treated with 32 mg/kg PB died within 2 days (*p* = 0.0052, compared with the HD5lp5 group). Pathological analysis revealed that compared with the control treatment, the PB treatment induced apparent renal tubular injury after 1.5 days (Figure 4D), which was supported by increased levels of blood urea nitrogen (BUN, Figure 4E) and creatinine (CRE, Figure 4F). In contrast, neither abnormal renal function indicators nor obvious pathological changes were detected in the mice treated with HD5lp5, suggesting that no overt toxicity occurred in vivo.

### 2.6. HD5lp5 Suppresses CRAB Infection In Vivo

**Figure 5:**
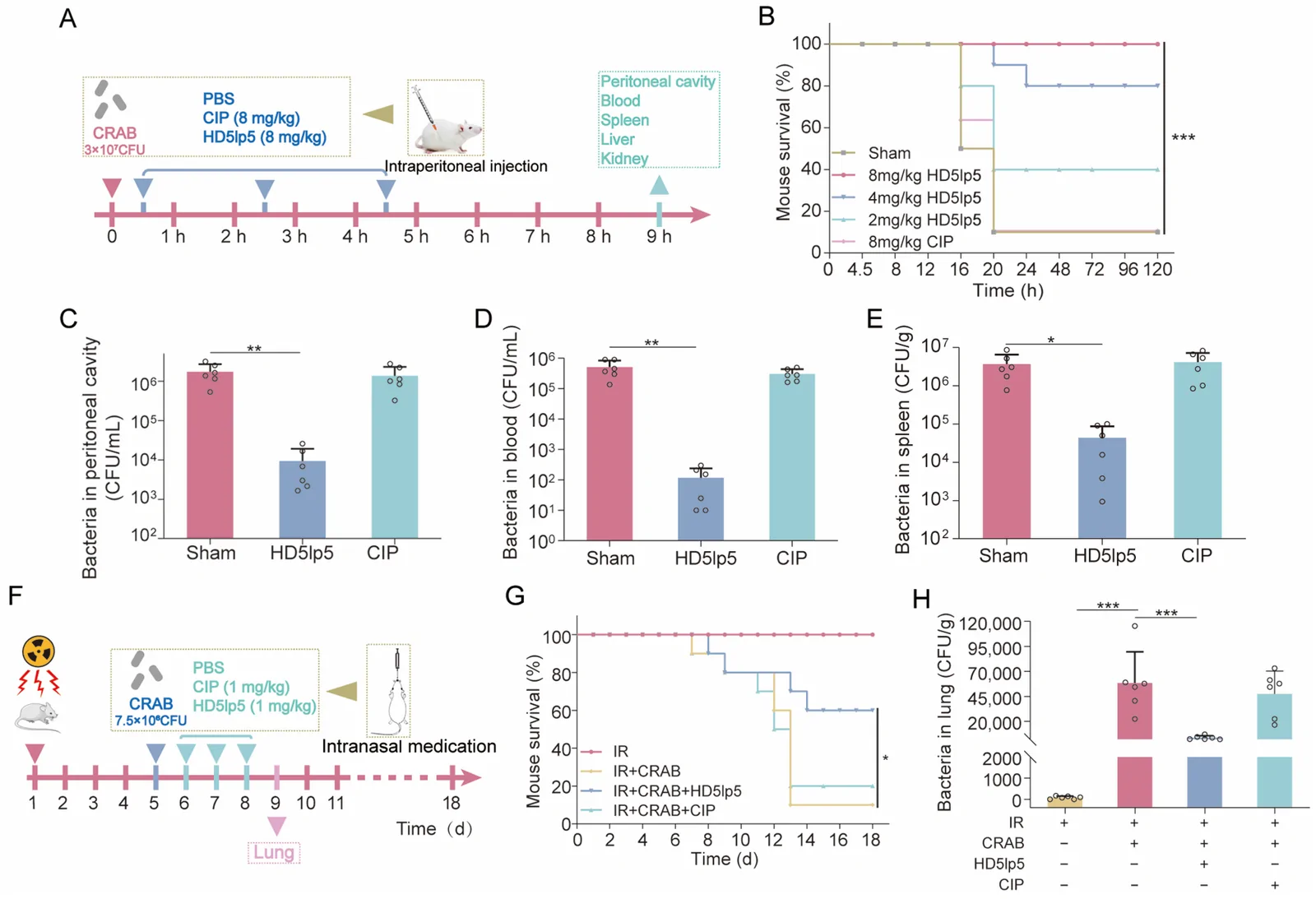
Antibacterial assessment of HD5lp5 against CRAB in vivo. (**A**) Experimental workflow of the intra-abdominal CRAB infection experiment in mice. (**B**) Survival of mice treated with HD5lp5 and CIP. Each group included ten mice. Survival curves were plotted using the Kaplan–Meier method. ***, *p* < 0.001 compared with the sham group, as determined by the log-rank test. (**C**) Bacterial counts in mouse peritoneal lavage fluid, (**D**) blood, and (**E**) spleen. The results are shown as the mean ± SD of six biological replicates (*n* = 6). *, *p <* 0.05; **, *p <* 0.01, as determined by one-way ANOVA followed by Tukey’s multiple comparisons test. (**F**) Experimental workflow of irradiation-assisted local pulmonary CRAB infection in mice. (**G**) Survival of mice treated with HD5lp5 and CIP. Each group included ten mice. Survival curves were plotted using the Kaplan–Meier method. *, *p* < 0.05 compared with the irradiation (IR) + CRAB group, as determined by the log-rank test. (**H**) Bacterial counts in mouse lungs. The results are shown as the mean ± SD of six biological replicates (*n* = 6). ***, *p* < 0.001, as determined by one-way ANOVA followed by Tukey’s multiple comparisons test.

To evaluate the therapeutic potential of HD5lp5, we established a murine model of abdominal sepsis induced by CRAB (Figure 5A), in which BALB/c mice were challenged intraperitoneally with 3 × 10^7^ CFU of the bacteria. Different doses of HD5lp5 (2, 4, and 8 mg/kg) were subsequently administered at 0.5, 2.5, and 4.5 h after bacterial injection, according to established protocols [36]. A separate group of infected mice treated with 8 mg/kg CIP was used to confirm the resistance phenotype of the clinical isolate. Survival analysis revealed a 90% mortality rate within 5 days in untreated infected mice, with no improvement in survival observed following CIP treatment (Figure 5B). In marked contrast, HD5lp5 conferred substantial, dose-dependent protection, with the 8 mg/kg cohort surviving throughout the observation period (*p* = 0.0008, compared with the sham group). Corroborating these results, quantification of the bacterial burden at the 9-h time point revealed that HD5lp5 reduced the peritoneal bacterial count to 9.3 × 10^3^ CFU (Figure 5C), which was significantly lower than that measured in the sham group (1.73 × 10^6^ CFU). This potent bactericidal activity was also evident in the bloodstream (Figure 5D), spleen (Figure 5E), kidney, and liver (Supplementary Figure S3) of mice treated with HD5lp5.

Having established its efficacy systemically, we next investigated whether HD5lp5 could function effectively as a topical antibiotic in a pulmonary model. Recognising that CRAB infections predominantly afflict immunocompromised patients [37], we used a single 5 Gy dose of γ-irradiation to ablate the murine immune system as previously described [19,22], and then administered an intranasal CRAB challenge (7.5 × 10^6^ CFU) four days post-irradiation (Figure 5F). Twenty-four hours after infection, HD5lp5 (1 mg/kg in 50 μL) was administered intranasally once daily for three consecutive days. As demonstrated in Figure 5G, 90% of the immunocompromised and infected mice in the control group died within two weeks. Notably, whereas CIP offered no survival benefit, HD5lp5 treatment increased the survival rate to 60% (*p* = 0.0321, compared with the IR + CRAB group). Consistent with the survival data, the lung bacterial load in the HD5lp5-treated mice on day 9 was 3.67 × 10^3^ CFU/g (Figure 5H), which was dramatically lower than that in the sham group (5.83 × 10^4^ CFU/g). In conclusion, these results collectively establish that HD5lp5 is a potent anti-CRAB agent that is effective in both systemic and topical applications.

## 3. Conclusions

**Figure 6:**
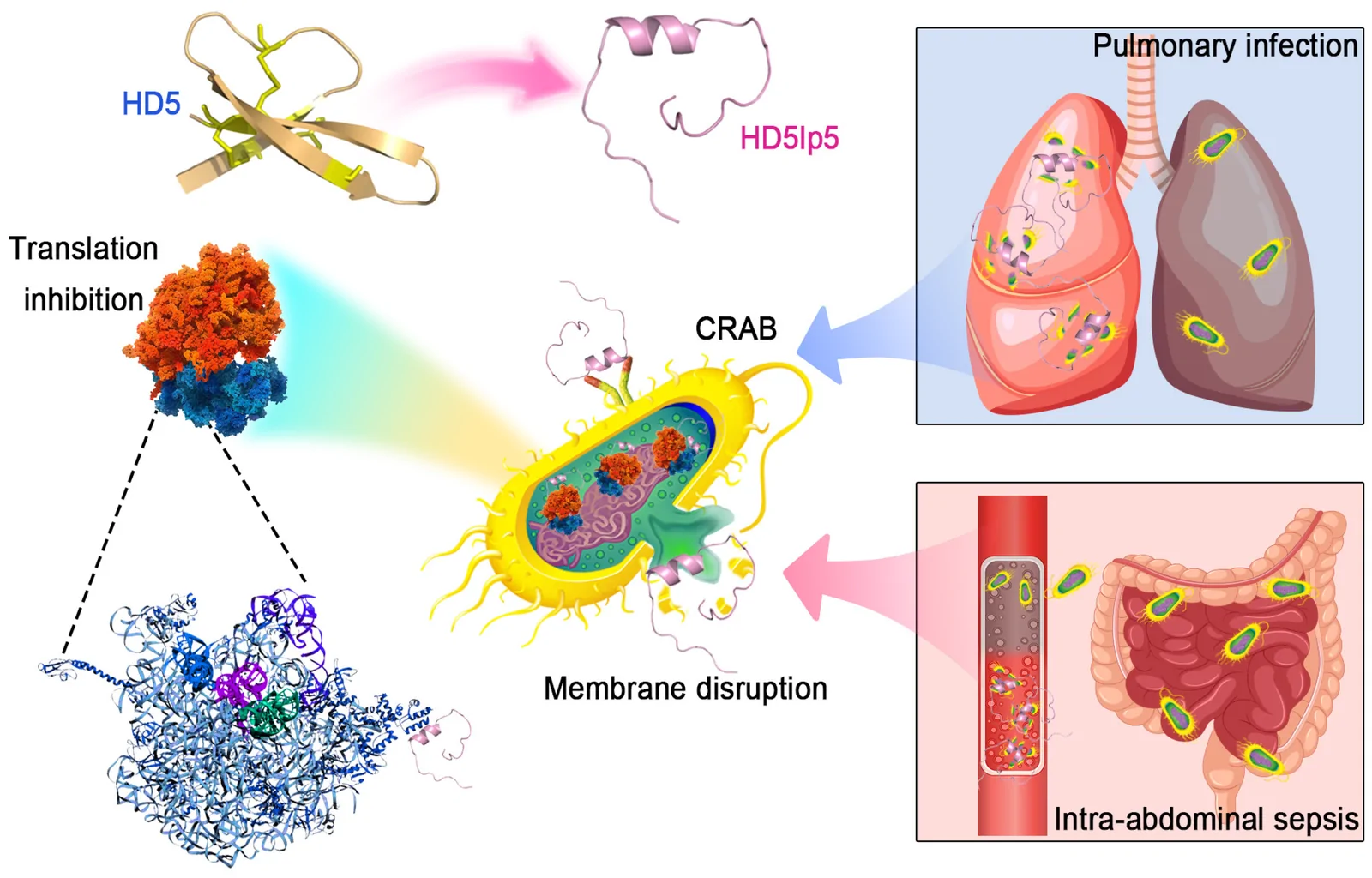
Schematic illustration of the design and application of HD5lp5 as a preclinical candidate to treat CRAB infections.

CRAB is among the top priority pathogens targeted for the development of novel therapeutics. In this study, we designed a linear AMP derived from the human intestinal defensin HD5 by removing its disulfide bonds and incorporating Arg residues. The resulting derivative, termed HD5lp5, exhibits rapid and potent antibacterial activity against CRAB and retains its bioactivity even in complex body fluid environments. Mechanistically, HD5lp5 binds with high affinity to the bacterial membrane component lipid A and disrupts membrane integrity in a dose-dependent manner (Figure 6). Furthermore, HD5lp5 exhibits an inhibitory effect on ribosomal translation in vitro, suggesting a potential ribosomal-targeting mechanism underlying its antibacterial activity. Accordingly, HD5lp5 is effective at eliminating CRAB in murine models of intra-abdominal sepsis and irradiation-assisted local pulmonary infection, significantly improving mouse survival.

Despite the promising preclinical results, our study has several limitations. First, the number of clinical CRAB isolates tested was limited and may not represent the full diversity of clinical strains. Future studies should collect additional CRAB isolates exhibiting diverse resistance phenotypes to further establish the antibacterial efficacy of HD5lp5 across the broader CRAB population. Second, although HD5lp5 was shown to bind the ribosomal L7/L12 protein and inhibit translation in vitro, whether L7/L12 actually mediates this inhibition and the underlying mechanism remain to be determined. Third, toxicity testing was limited in scope, and the lack of apparent toxicity in the present models does not rule out adverse effects under other conditions. Fourth, the efficacy observed in murine models of intra-abdominal sepsis and irradiation-assisted pulmonary infection cannot be directly extrapolated to human CRAB infection. Given that the in vivo experiments were designed as exploratory proof-of-concept studies, no formal sample-size calculation was performed; therefore, the findings of this study are preliminary and require validation in larger, adequately powered studies. Moreover, the pharmacokinetics, immunogenicity, repeated-dose toxicity, tissue distribution, stability, and therapeutic index of HD5lp5 were not comprehensively assessed. These issues should be addressed in future studies before the clinical application of HD5lp5 can be advanced.

## 4. Materials and Methods

### 4.1. Materials

Mueller–Hinton broth (MHB) medium was purchased from Solarbio (M8556; Beijing, China). HD5 was obtained from MCE (HY-P5694, Shanghai, China). Lipoteichoic acid (LTA; L2515), lipid A (L5399), bovine serum albumin (BSA; A1933), sulfo-N-hydroxysuccinimide (s-NHS; 56485), 1-ethyl-3-(3-dimethylaminopropyl)carbodiimide hydrochloride (EDC; E1769), and *N*-phenyl-1-naphthylamine (NPN; 104043) were obtained from Sigma-Aldrich (St. Louis, MO, USA). Proteinase K (ST535), phenylmethylsulfonyl fluoride (PMSF; ST506), and *o*-nitrophenyl-β-D-galactoside (ONPG; ST429) were purchased from Beyotime (Shanghai, China). Amino-reactive second-generation (AR2G) and streptavidin (SA) biosensors were obtained from Sartorius (Göttingen, Germany). Acetonitrile (ACN; 4340863), acetic acid (984303), trifluoroacetic acid (TFA; 28901), FM4–64 (F34653), high-glucose DMEM, and foetal bovine serum (FBS) were purchased from Thermo Fisher Scientific (Shanghai, China). A Cell Counting Kit (CCK)-8 was obtained from Dojindo (CK04; Shanghai, China).

### 4.2. Peptide Synthesis

HD5lps were synthesised by Guoping Pharmaceutical (Hefei, China) using automated solid-phase protocols. Purity analysis was carried out on a Shimadzu (Kyoto, Japan) LC-10AT HPLC system fitted with an Inertsil ODS-SP column (5 μm, 4.6 × 250 mm; GL Sciences, Kyoto, Japan), with a linear gradient from 15% to 55% mobile phase B (0.1% TFA in ACN) over mobile phase A (0.1% TFA in water). Mass confirmation was performed on a Shimadzu LCMS-2020 mass spectrometer. The corresponding HPLC traces and mass spectra, in which the measured molecular weights agree closely with the calculated values, are shown in Supplementary Figure S4.

### 4.3. Bacterial Strains

The four standard strains used for antibacterial evaluation, *A. baumannii* (strain no. 17978), *P. aeruginosa* (PAO1), *S. aureus* (strain no. 25923), and methicillin-resistant *S. aureus* (MRSA; strain no. 43300), were obtained from the American Type Culture Collection (ATCC). CRAB-*No. 1* was clinically isolated and identified by an API 20NE system (bioM′erieux, Marcy l’Etoile, FRA), with its antibiotic resistance (resistant to: doxycycline, piperacillin/tazobactam, cotrimoxazole, levofloxacin, CIP, tobramycin, meropenem, imipenem, cefepime, ceftazidime, and cefoperazone/sulbactam; susceptible to: minocycline, tigecycline, and colistin) verified via VITEK-2 compact system analysis (bioM′erieux) [19].

### 4.4. Antibacterial Assay

The bacteria were cultured in MHB at 180 rpm and 37 °C to the mid-log phase and diluted to 2 × 10^5^ CFU/mL. Peptides were prepared in 0.01% acetic acid solution containing 0.2% BSA to final concentrations of 6400, 3200, 1600, 800, 400, 200, 100, 50, and 25 μg/mL. Peptide MICs were determined using the broth microdilution procedure, following CLSI guidelines with modifications established by the Hancock laboratory [38] (https://cmdr.ubc.ca/bobh/method/modified-mic-method-for-cationic-antimicrobial-peptides/; (accessed on 16 September 2026)). In sterile polypropylene microtiter plates (3365; Corning, New York, NY, USA), 100 μL of bacterial suspension in MHB was mixed with 11 μL of peptide solution. The optical density at 600 nm was measured following an 18-h incubation at 37 °C. The MIC was defined as the lowest peptide concentration that reduced bacterial turbidity by 70%. The 70% threshold was established to minimize false-negative results arising from bacterial aggregation induced by high concentrations of cationic peptides. The MIC assay was repeated three times.

To evaluate the salt-resistant antibacterial action of the peptides, CRAB was cultured to the mid-log phase and adjusted to 1 × 10^6^ CFU/mL in 10 mM PBS (pH = 7.4) containing 1% MHB and increasing concentrations of NaCl (25, 75, and 150 mM). A total of 90 μL of bacterial suspension was mixed with 10 μL of 100 μg/mL peptide in sterile polypropylene microtiter plates, and coincubation was performed at 130 rpm and 37 °C for 1 h. Afterwards, the suspension was diluted and spread onto MHB plates, and bacterial colonies were counted after culture in a constant temperature incubator for 18 h. To evaluate the antibacterial activity in the presence of serum, 10 μL of 250 μg/mL peptides was incubated with 90 μL of 1 × 10^6^ CFU/mL CRAB in 10 mM PBS supplemented with 20% fresh mouse serum, which was obtained by centrifuging the whole blood at 3000 rpm for 10 min. Coincubation was carried out at 37 °C for 5 and 10 min, and bacterial culture and counting were performed as mentioned above. These experiments were repeated three times.

### 4.5. BLI

The binding of HD5lp5 to lipid A, LTA, and RplL was measured using Forte Bio’s “Octet Red 96” BLI system (Sartorius). AR2G sensors were equilibrated in ultrapure water at room temperature for 10 min and activated for 300 s with a mixture of 20 mM EDC and 10 mM s-NHS. Lipid A and LTA (20 μg/mL, pH 5.0) were then immobilised onto the biosensors for 300 s at 600 rpm and 30 °C, followed by termination with 1 M ethanolamine solution (pH 8.5). HD5lp5 was prepared in 10 mM PBS at increasing concentrations. Responses for the association and dissociation were measured under the same conditions for 300 s [17]. To investigate the association of HD5lp5 with L7/L12 protein, the protein (100 µg/mL, 100 µL) prepared via a recombinant expression system in *E. coli* (Detai Bioengineering, Nanjing, China) was incubated with 100 μM biotin (Genemore, Shanghai, China) in the dark for 30 min. Biotinylated L7/L12 protein was then collected by Zeba^TM^ Spin desalting columns (Thermo Fisher Scientific, Shanghai, China) and immobilised on streptavidin biosensors (Sartorius). The results were collected and processed using Fortebio Data Analysis 7.0 software. The equilibrium K_D_ was generated using a 1:1 binding model. The BLI assay was repeated twice.

### 4.6. Outer Membrane Penetration Assay

CRAB was grown to the mid-log phase and then adjusted to 1 × 10^8^ CFU/mL in 10 mM PBS supplemented with 1% MHB. Aliquots of 190 μL of this suspension were mixed with 10 μL of peptides at 31.25, 62.5, and 125 µg/mL, and incubated at 37 °C with shaking at 130 rpm for 1 h. Subsequently, NPN was introduced at a final concentration of 10 μM. Fluorescence was measured with a Tecan Infinite M1000 Pro microplate reader (Männedorf, Zürich, Switzerland) at an emission wavelength of 408 nm, following excitation at 350 nm. This assay was conducted in duplicate and repeated three times.

### 4.7. Inner Membrane Permeabilization Assay

Inner membrane disruption by HD5 and HD5lp5 was assessed with *E. coli* ML35 (ATCC 43827), which lacks lactose permease yet constitutively produces cytosolic β-galactosidase. Once the inner membrane is permeabilized, the liberated enzyme converts the chromogenic substrate ONPG to ONP, which is detectable spectrophotometrically at 405 nm. *E. coli* ML35 was cultured to mid-log phase in nutrient broth (234000; BD, Franklin Lakes, NJ, USA) and then diluted to 1 × 10^7^ CFU/mL in sterile 10 mM PBS supplemented with 1% tryptic soytone broth (TSB; 211825, BD). HD5 was dissolved in sterile water to concentrations of 30 and 60 µg/mL, while HD5lp5 was prepared similarly at 7.5 and 15 µg/mL. On microtiter plates, 80 μL of bacterial suspension was combined with 10 μL of ONPG (25 mM) and 10 μL of peptide solution or sterile water. The plates were incubated at 37 °C for 2 h with 50 s of shaking every 5 min, after which the absorbance was recorded at 5 min intervals. This experiment was repeated three times.

### 4.8. SEM

CRAB was harvested at mid-log phase from MHB cultures and resuspended in sterile 10 mM PBS to a density of 2 × 10^9^ CFU/mL. Peptide treatment was carried out by mixing 100 μL of the bacterial suspension with 100 μg/mL HD5 or HD5lp5, followed by incubation at 37 °C with agitation at 130 rpm for either 15 or 30 min. A 10 μL drop of each sample was applied to poly-L-lysine-coated coverslips and air-dried at room temperature. Fixation was performed overnight with 2.5% glutaraldehyde at 4 °C; the samples were then passed through a graded ethanol series and dried with tert-butanol. Imaging of bacterial morphology was conducted on a Crossbeam 340 scanning electron microscope (Zeiss, Oberkochen, Germany).

### 4.9. Circular Dichroism (CD) Spectroscopy

HD5lp5 was dissolved in sterile water to a concentration of 100 μg/mL, with or without 20 mM sodium dodecyl sulfate (SDS); the latter served to mimic the prokaryotic membrane. CD spectra were acquired at 25 °C on a Chirascan spectrometer (Applied Photophysics; Leatherhead, Surrey, UK) using a 0.5 mm quartz cuvette. Measurements spanned 190–260 nm with a data pitch of 1 nm and a dwell time of 0.5 s, yielding a total acquisition time of approximately 73 s per scan. Data from three independent experiments were averaged and corrected with Pro-Data Chirascan (v4.1).

### 4.10. Confocal Microscopy

To assess peptide translocation into CRAB, mid-log-phase bacteria were adjusted to 2 × 10^8^ CFU/mL in sterile 10 mM PBS. The bacterial membranes were counterstained with 8 µM FM4-64 on ice for 2 min, after which the unbound dye was removed by centrifugation (9000 rpm, 4 °C, 5 min). Then, the pellet was resuspended in 100 μL of PBS containing 1% MHB, followed by the addition of 10 μL of 25 μg/mL FITC-HD5lp5. Following coincubation at 37 °C and 130 rpm for 15 and 30 min, aliquots were mounted on poly-L-lysine-coated coverslips, air-dried, and fixed with 4% paraformaldehyde. Peptide internalisation was visualised by confocal microscopy (LSM800, Zeiss, Oberkochen, Germany). 

### 4.11. Identification of Internal Targets of HD5lp5 in Bacteria

*A. baumannii* (ATCC 17978) was cultured in MHB to mid-log phase and adjusted to 2 × 10^8^ CFU/mL in sterile 10 mM PBS. A 1-mL aliquot of this suspension was coincubated with 25 µg/mL biotin-HD5lp5 or HD5lp5 at 37 °C and 130 rpm for 2 h. Unlabelled HD5lp5 was used as the control group. After centrifugation at 4 °C and 9000 rpm for 5 min, the pellet was resuspended in 100 µL of sterile water. Bacteria were then lysed by ultrasonication on ice, and the lysate was collected by centrifugation at 10,000 rpm and 4 °C for 10 min. Streptavidin-coated magnetic beads (Dynabeads^TM^ M-280, Thermo Fisher Scientific, Shanghai, China) preequilibrated with PBS containing 0.1% Tween-20 were then added, and the mixture was incubated at room temperature for 2 h. Subsequently, the beads were washed extensively with PBS containing 0.1% Tween-20 and 500 mM NaCl to remove nonspecific binders. Bound proteins were eluted by boiling in SDS loading buffer, resolved by SDS–PAGE, and subjected to in-gel trypsin digestion. The resulting peptides were analysed by liquid chromatography (LC)-mass spectrometry (MS) on an LTQ Orbitrap Velos Pro system (Thermo Fisher Scientific) coupled with a U3000 nanoLC (Thermo Fisher Scientific, Shanghai, China). Peptides were separated on a C18 column (75 μm × 15 cm) using a 106-min gradient of 0.1% formic acid (A) and 80% acetonitrile in 0.1% formic acid (B) at 400 nL/min. The mass spectrometer was operated in DDA mode, with full MS scans (*m*/*z* 350–1800, resolution 60,000). Raw data were searched against the *A. baumannii* ATCC 17978 database using Proteome Discoverer. The precursor and fragment ion mass tolerances were set to 10 ppm and 0.02 Da, respectively. The peptide-spectrum matches were filtered using a target-decoy strategy to ensure a false discovery rate (FDR) of <1% at the peptide and protein levels. Proteins identified by at least two unique peptides were considered reliable. The experiment was performed in three independent biological replicates. Proteins that were captured in at least two of the three replicates in the biotin-HD5lp5-treated group but not in the control group, with a score > 0, were considered potential targets.

### 4.12. In Vitro Translation Assay

The inhibitory activity of HD5lp5 against the bacterial 70S ribosome was evaluated using a PURExpress in vitro protein synthesis system (NEB, Ipswich, MA, USA). Each 5-µL reaction contained 2 µL of Solution A, 0.6 µL of Factor Mix, 0.9 µL of 70S ribosomes, 0.8 µL of DNA plasmid, and varying concentrations of the test compound. HD5lp5 was added at final concentrations of 0.01, 0.1, 1, 10, 100, and 1000 µM. Reactions were incubated at 37 °C for 4 h in triplicate and then terminated on ice. The protein yield in each reaction was determined using a BCA assay and corrected by subtracting the background signal from the control reactions lacking the DNA template. The half-maximal inhibitory concentration (IC_50_) was obtained by fitting the dose–response data via nonlinear regression. This experiment was conducted in duplicate and repeated three times.

### 4.13. Toxicological Evaluation

The haemolytic activity of HD5lp5 was assessed as previously described [7]. A 300 μL portion of mouse red blood cells suspended in 0.9% NaCl was combined with 1.2 mL of HD5lp5 at 50 or 100 μg/mL and incubated at 37 °C for 2 h. The negative control consisted of HD5 at the same concentrations, whereas melittin at 12.5 and 25 μg/mL served as the positive control. After centrifugation at 10,000 rpm for 5 min, the absorbance of the supernatant was determined at 570 nm. The extent of haemolysis was determined as (A_pep_ − A_blank_)/(A_tot_ − A_blank_) × 100%, in which A_pep_, A_blank_, and A_tot_ are the absorbances of the peptide-treated sample, the negative control, and the Triton X-100-treated positive control, respectively. Cytotoxicity towards HUVECs (CAS, Shanghai, China), which were seeded at 1 × 10^4^ cells per well in 96-well plates and maintained in DMEM supplemented with 10% FBS, was evaluated after the cells reached adherence by exposing them to 50 or 100 μg/mL peptide for 24 h at 37 °C, followed by CCK-8 measurement of cell viability. The assays were conducted in duplicate and repeated three times.

To assess in vivo acute toxicity, 60 BALB/c mice (8 weeks old, 18–22 g) were randomly allocated to 12 groups (*n* = 5) using a standard random number table. Each animal was assigned a sequential ID, and group allocation was determined by reading digits from the table. The mice received a single intraperitoneal injection of HD5lp5 or PB at concentrations of 8, 16, 32, 64, 128, and 256 mg/kg. Mouse survival was monitored for 5 days. To evaluate the influence of continued HD5lp5 administration, another 20 mice were randomly divided into two groups (*n* = 10) using a standard random number table and were intraperitoneally administered 64 mg/kg HD5lp5 or 32 mg/kg PB twice daily. The sample size was based on our previous experience with this model [39], in which ten mice per group were sufficient to detect consistent and reproducible differences. The survival of the mice was recorded over a 4-day period. For pathological analysis, mouse blood samples and major organs, including the liver, kidney, and spleen, were collected. BUN and CRE levels were measured using a Beckman (Brea, CA, USA) AU5800 automatic biochemical analyser. The tissue samples were fixed in 4% paraformaldehyde at 4 °C for 48 h, dehydrated through a graded ethanol series, and embedded in paraffin, after which 3-μm sections were prepared and stained with haematoxylin and eosin (HE). Histopathological evaluation was performed by a board-certified pathologist who was blinded to group allocation. Samples were coded with numeric identifiers before assessment. No a priori exclusion criteria were set. All animals that received the allocated intervention were included in the analysis. All the animal procedures were approved by the Animal Experimental Ethics Committee of AMU (AMUWEC20230331) and conducted in compliance with the NIH Guidelines for the Care and Use of Laboratory Animals (NIH publication no. 85–23, rev. 1985).

### 4.14. Intra-Abdominal CRAB Infection Experiment

Fifty BALB/c mice (8 weeks old, 18–22 g) were randomly assigned to five groups (*n* = 10) as described in the toxicological evaluation section. The mice received an intraperitoneal injection of 3 × 10^7^ CFU of CRAB to establish bacterial infection. Increasing concentrations of HD5lp5 (2, 4, and 8 mg/kg) and 8 mg/kg CIP were administered in the same manner at 0.5, 2.5, and 4.5 h after infection, as described in a previous study [36]. The survival of the mice was monitored for 5 days. To evaluate in vivo antibacterial efficacy, an independent cohort of 18 mice was randomised into three groups (*n* = 6): sterile PBS (sham), 8 mg/kg HD5lp5, and 8 mg/kg CIP. The mice were euthanised at 9 h postinfection, and the peritoneal lavage fluid, blood, spleen, liver, and kidney were collected. The samples were processed with or without homogenisation in PBS and plated on MHB agar, and the bacterial burden was quantified after 18 h at 37 °C.

### 4.15. Irradiation-Assisted Pulmonary CRAB Infection Experiment

Forty BALB/c mice (8 weeks old, 18–22 g) were randomised into four groups (*n* = 10) as described in the toxicological evaluation section. The mice were immunosuppressed by total-body γ-irradiation (5 Gy, ^60^Co source, 0.45 Gy/min). Mid-log-phase CRAB was harvested and then washed three times in sterile PBS, after which 7.5 × 10^6^ CFU was delivered intranasally to each mouse four days after irradiation. HD5lp5 and CIP were administered via the same route at 1 mg/kg once daily for three consecutive days 24 h after bacterial infection. Mouse survival was monitored for 18 days. To quantify bacterial loads in the lungs, mouse lung tissues were harvested four days after infection, weighed and homogenised in PBS, and further plated onto MHB agar. After 18 h of incubation at 37 °C, the resulting bacterial colonies were counted. Samples with no detectable colonies were recorded as 0.

### 4.16. Statistical Analysis

Statistical analysis was performed using GraphPad Prism 9.0 software. The effects of treatment (HD5 vs. HD5lp5) and concentration (or incubation time) on bacterial clearance were analysed by two-way ANOVA, followed by simple effects analysis using Sidak’s multiple comparisons test. For in vivo toxicity and bacterial clearance in organs, one-way ANOVA followed by Tukey’s multiple comparisons test was used. Normality and homogeneity of variance were assessed using the Shapiro–Wilk test and Levene’s test, respectively. Survival curves were generated using the Kaplan–Meier method, and differences in survival among groups were analysed by the log-rank test. A *p* value less than 0.05 was considered to indicate statistical significance.

## Data Availability

The original contributions presented in this study are included in the article. Further inquiries can be directed to the corresponding author.

## Supplementary Materials

The following supporting information can be downloaded at: https://www.mdpi.com/article/10.3390/molecules31213885/s1, Figure S1: Recombinant expression plasmid and SDS-PAGE analysis of ribosomal L7/L12 protein; Figure S2: Survival of HUVEC cells exposed to peptides after 24 h; Figure S3: Bacterial counts in mice kidney and liver; Figure S4: Detailed purities and MWs of synthetic peptides; Table S1: Amino acid sequences, charges, and GRAVY values of peptides; Table S2: Mass spectrometry analysis revealing six intracellular protein targets for HD5lp5.

## References

[1] Tacconelli E., Carrara E., Savoldi A., Harbarth S., Mendelson M., Monnet D.L., Pulcini C., Kahlmeter G., Kluytmans J., Carmeli Y. (2018). Discovery, Research, and Development of New Antibiotics: The WHO Priority List of Antibiotic-Resistant Bacteria and Tuberculosis. Lancet Infect. Dis..

[2] GBD 2021 Antimicrobial Resistance Collaborators (2024). Global Burden of Bacterial Antimicrobial Resistance 1990–2021: A Systematic Analysis with Forecasts to 2050. Lancet.

[3] Makwana N., Karad D.D., Yadav B., Saini C., Mercier C., Kharat A.S. (2026). Global Strategies to Fight Carbapenem-Resistant *Acinetobacter baumannii* (CRAB) Infections. Microb. Pathog..

[4] Sati H., Carrara E., Savoldi A., Hansen P., Garlasco J., Campagnaro E., Boccia S., Castillo-Polo J.A., Magrini E., Garcia-Vello P. (2025). The WHO Bacterial Priority Pathogens List 2024: A Prioritisation Study to Guide Research, Development, and Public Health Strategies Against Antimicrobial Resistance. Lancet Infect. Dis..

[5] Vila J., Moreno-Morales J., Ballesté-Delpierre C. (2020). Current Landscape in the Discovery of Novel Antibacterial Agents. Clin. Microbiol. Infect..

[6] Magana M., Pushpanathan M., Santos A.L., Leanse L., Fernandez M., Ioannidis A., Giulianotti M.A., Apidianakis Y., Bradfute S., Ferguson A.L. (2020). The Value of Antimicrobial Peptides in the Age of Resistance. Lancet Infect. Dis..

[7] Zhao G., Jia C., Zhu C., Fang M., Li C., Chen Y., He Y., Han S., He Y., Gao J. (2022). γ-Core Guided Antibiotic Design Based on Human Enteric Defensin 5. Membranes.

[8] Gao X., Ding J., Liao C., Xu J., Liu X., Lu W. (2021). Defensins: The Natural Peptide Antibiotic. Adv. Drug Deliv. Rev..

[9] Shafee T.M., Lay F.T., Phan T.K., Anderson M.A., Hulett M.D. (2017). Convergent Evolution of Defensin Sequence, Structure and Function. Cell. Mol. Life Sci..

[10] Ericksen B., Wu Z., Lu W., Lehrer R.I. (2005). Antibacterial Activity and Specificity of the Six Human Alpha-Defensins. Antimicrob. Agents Chemother..

[11] Tasdemiroglu Y., Gourdie R.G., He J.Q. (2022). In Vivo Degradation Forms, Anti-Degradation Strategies, and Clinical Applications of Therapeutic Peptides in Non-Infectious Chronic Diseases. Eur. J. Pharmacol..

[12] Zorzi A., Middendorp S.J., Wilbs J., Deyle K., Heinis C. (2017). Acylated Heptapeptide Binds Albumin with High Affinity and Application As Tag Furnishes Long-Acting Peptides. Nat. Commun..

[13] de Leeuw E., Rajabi M., Zou G., Pazgier M., Lu W. (2009). Selective Arginines Are Important for the Antibacterial Activity and Host Cell Interaction of Human Alpha-Defensin 5. FEBS Lett..

[14] Lehrer R.I., Jung G., Ruchala P., Andre S., Gabius H.J., Lu W. (2009). Multivalent Binding of Carbohydrates by the Human Alpha-Defensin, HD5. J. Immunol..

[15] Jobin M.L., Alves I.D. (2014). On the Importance of Electrostatic Interactions Between Cell Penetrating Peptides and Membranes: A Pathway Toward Tumor Cell Selectivity?. Biochimie.

[16] Frecer V., Ho B., Ding J.L. (2004). De Novo Design of Potent Antimicrobial Peptides. Antimicrob. Agents Chemother..

[17] Wang C., Shen M., Zhang N., Wang S., Xu Y., Chen S., Chen F., Yang K., He T., Wang A. (2016). Reduction Impairs the Antibacterial Activity but Benefits the LPS Neutralization Ability of Human Enteric Defensin 5. Sci. Rep..

[18] Bucataru C., Ciobanasu C. (2024). Antimicrobial Peptides: Opportunities and Challenges in Overcoming Resistance. Microbiol. Res..

[19] Jiang Y., Zhao G., Gong Y., Chen Y., Li C., Han S., Deng Y., Zhao J., Wang J., Wang C. (2025). Dodecapeptides Derived from Human Cathelicidin with Potent Activity Against Carbapenem-Resistant *Acinetobacter baumannii*. Eur. J. Med. Chem..

[20] Wang C., Shen M., Gohain N., Tolbert W.D., Chen F., Zhang N., Yang K., Wang A., Su Y., Cheng T. (2015). Design of a Potent Antibiotic Peptide Based on the Active Region of Human Defensin 5. J. Med. Chem..

[21] Gao C., Wang Y., Zhang Y., Wei J., Cheng X., Zhang J., Zou Q., Gu J. (2020). Complete Genome Sequence of *Pseudomonas aeruginosa* XN-1, Isolated From the Sputum of a Severe Pneumonia Patient. Microbiol. Resour. Announc..

[22] Wang C., Zhao G., Wang S., Chen Y., Gong Y., Chen S., Xu Y., Hu M., Wang X., Zeng H. (2018). A Simplified Derivative of Human Defensin 5 with Potent and Efficient Activity Against Multidrug-Resistant *Acinetobacter baumannii*. Antimicrob. Agents Chemother..

[23] Maisetta G., Di Luca M., Esin S., Florio W., Brancatisano F.L., Bottai D., Campa M., Batoni G. (2008). Evaluation of the Inhibitory Effects of Human Serum Components on Bactericidal Activity of Human Beta Defensin 3. Peptides.

[24] Mourtada R., Herce H.D., Yin D.J., Moroco J.A., Wales T.E., Engen J.R., Walensky L.D. (2019). Design of Stapled Antimicrobial Peptides That Are Stable, Nontoxic and Kill Antibiotic-Resistant Bacteria in Mice. Nat. Biotechnol..

[25] Chen F., Tang Y., Zheng H., Xu Y., Wang J., Wang C. (2019). Roles of the Conserved Amino Acid Residues in Reduced Human Defensin 5: Cysteine and Arginine Are Indispensable for Its Antibacterial Action and LPS Neutralization. ChemMedChem.

[26] Schmidt N.W., Tai K.P., Kamdar K., Mishra A., Lai G.H., Zhao K., Ouellette A.J., Wong G.C. (2012). Arginine in α-Defensins: Differential Effects on Bactericidal Activity Correspond to Geometry of Membrane Curvature Generation and Peptide-Lipid Phase Behavior. J. Biol. Chem..

[27] Wilson D.N. (2014). Ribosome-Targeting Antibiotics and Mechanisms of Bacterial Resistance. Nat. Rev. Microbiol..

[28] Lin J., Zhou D., Steitz T.A., Polikanov Y.S., Gagnon M.G. (2018). Ribosome-Targeting Antibiotics: Modes of Action, Mechanisms of Resistance, and Implications for Drug Design. Annu. Rev. Biochem..

[29] Schwarz S., Shen J., Kadlec K., Wang Y., Brenner Michael G., Feßler A.T., Vester B. (2016). Lincosamides, Streptogramins, Phenicols, and Pleuromutilins: Mode of Action and Mechanisms of Resistance. Cold Spring Harb. Perspect. Med..

[30] Diaconu M., Kothe U., Schlünzen F., Fischer N., Harms J.M., Tonevitsky A.G., Stark H., Rodnina M.V., Wahl M.C. (2005). Structural Basis for the Function of the Ribosomal L7/12 Stalk in Factor Binding and GTPase Activation. Cell.

[31] Kavran J.M., Steitz T.A. (2007). Structure of the Base of the L7/L12 Stalk of the *Haloarcula marismortui* Large Ribosomal Subunit: Analysis of L11 Movements. J. Mol. Biol..

[32] Godinic-Mikulcic V., Jaric J., Greber B.J., Franke V., Hodnik V., Anderluh G., Ban N., Weygand-Durasevic I. (2014). Archaeal Aminoacyl-tRNA Synthetases Interact With the Ribosome to Recycle tRNAs. Nucleic Acids Res..

[33] Bailly C. (2022). The Bacterial Thiopeptide Thiostrepton: An Update of Its Mode of Action, Pharmacological Properties and Applications. Eur. J. Pharmacol..

[34] Budagavi D.P., Chugh A. (2018). Antibacterial Properties of Latarcin 1 Derived Cell-Penetrating Peptides. Eur. J. Pharm. Sci..

[35] Paray B.A., Ahmad A., Khan J.M., Taufiq F., Pathan A., Malik A., Ahmed M.Z. (2021). The Role of the Multifunctional Antimicrobial Peptide Melittin in Gene Delivery. Drug Discov. Today.

[36] Nagarajan D., Roy N., Kulkarni O., Nanajkar N., Datey A., Ravichandran S., Thakur C., Thirunavukkarasu S., Aprameya I.V., Sarma S.P. (2019). Ω76: A Designed Antimicrobial Peptide to Combat Carbapenem- and Tigecycline-Resistant *Acinetobacter baumannii*. Sci. Adv..

[37] Müller C., Reuter S., Wille J., Xanthopoulou K., Stefanik D., Grundmann H., Higgins Paul G., Seifert H. (2023). A Global View on Carbapenem-Resistant *Acinetobacter baumannii*. mBio.

[38] Wu M., Hancock R.E. (1999). Interaction of the Cyclic Antimicrobial Cationic Peptide Bactenecin with the Outer and Cytoplasmic Membrane. J. Biol. Chem..

[39] Dong R., Liu R., Liu Z., Liu Y., Zhao G., Li H., Hou S., Ma X., Kang H., Liu J. (2025). Exploring the Repository of De Novo-Designed Bifunctional Antimicrobial Peptides Through Deep Learning. eLife.

