# Supplementary material for: Design of a Human Defensin 5-Derived Linear Peptide with Potent Antibacterial Efficacy Against Carbapenem-Resistant Acinetobacter baumannii
Source: Molecules. 2026 Oct 28;31(21):3885. doi: 10.3390/molecules31213885 (PMC13764960; doi:10.3390/molecules31213885)
Supplement: Supplementary file 1 [file molecules-31-03885-s001.zip › molecules-4603305-supplementary.pdf]

Supplementary Material

Design of a Human Defensin 5-Derived Linear Peptide with Potent Antibacterial Efficacy against Carbapenem-Resistant *Acinetobacter baumannii*

Gaomei Zhao[a#], Chengzhang Tan[a#], Xin Li[a#], Chenwenya Li[a], Mingrui Shu[b], Chao Wang[b], Kainuo Quan[b], Yongwu He[a], Jining Gao[a], Songling Han[a,*], Junping Wang[a,*], Cheng Wang[a,*]

[a] State Key Laboratory of Trauma and Chemical Poisoning, Institute of Combined Injury of PLA, College of Preventive Medicine, Army Medical University, Chongqing, 400038, China.

[b]College of Basic Medicine, Army Medical University, Chongqing, 400038, China.

Number of tables: 2

Number of figures: 4

Contents:

Table S1. Amino acid sequences, charges, and GRAVY values of peptides.

Table S2. Mass spectrometry analysis revealing six intracellular protein targets for HD5lp5.

Figure S1. Recombinant expression plasmid and SDS-PAGE analysis of ribosomal L7/L12 protein.

Figure S2. Survival of HUVEC cells exposed to peptides after 24 h.

Figure S3. Bacterial counts in mice kidney and liver.

Figure S4. Detailed purities and MWs of synthetic peptides.

Table S1. Amino acid sequences, charges, and GRAVY values of peptides

Peptide	Sequence	Charge	GRAVY[a]
HD5	ATCYCRTGRCATRESLSGVCEISGRLYRLCCR	+4	-0.113
HD5lp1	ATAYARTGRAATRESLSGVAEISGRLYRLAAR	+4	-0.244
HD5lp2	ATAYARTGRAATRRSLSGVARISGRLYRLAAR	+8	-0.306
HD5lp3	ATAYARTGRAATRRRLRGVARIRGRLYRLAAR	+11	-0.653
HD5lp4	ARAYARRGRAARRRRLRGVARIRGRLYRLAAR	+14	-1.009
HD5lp5	ARARARRGRAARRRRLRGVARIRGRLYRLAAR	+15	-1.109
HD5lp6	ARARARRGRAARRRRLRGVARIRGRLRRLAAR	+16	-1.209

[a]GRAVY was determined by ProtParam (https://web.expasy.org/protparam/). A lower value indicates a more hydrophilic property.

Table S2. Mass spectrometry analysis revealing six intracellular protein targets for HD5lp5

Accession	Description	Score	Area	MW (kD)
A3M1G2	50S ribosomal protein L7/L12 OS=*Acinetobacter baumannii*	21.18	5.604E6	12.7
A3M1F9	50S ribosomal protein L11 OS=*Acinetobacter baumannii*	14.17	6.237E6	15.0
A3M1G1	50S ribosomal protein L10 OS=*Acinetobacter baumannii*	15.03	1.130E6	18.1
A3M8W9	Chaperone protein DnaK OS=*Acinetobacter baumannii*	23.70	8.912E5	69.4
A3M1G0	50S ribosomal protein L1 OS=*Acinetobacter baumannii*	5.33	2.891E5	23.8
A3M237	Protein-export protein SecB OS=*Acinetobacter baumannii*	6.00	9.224E5	17.0

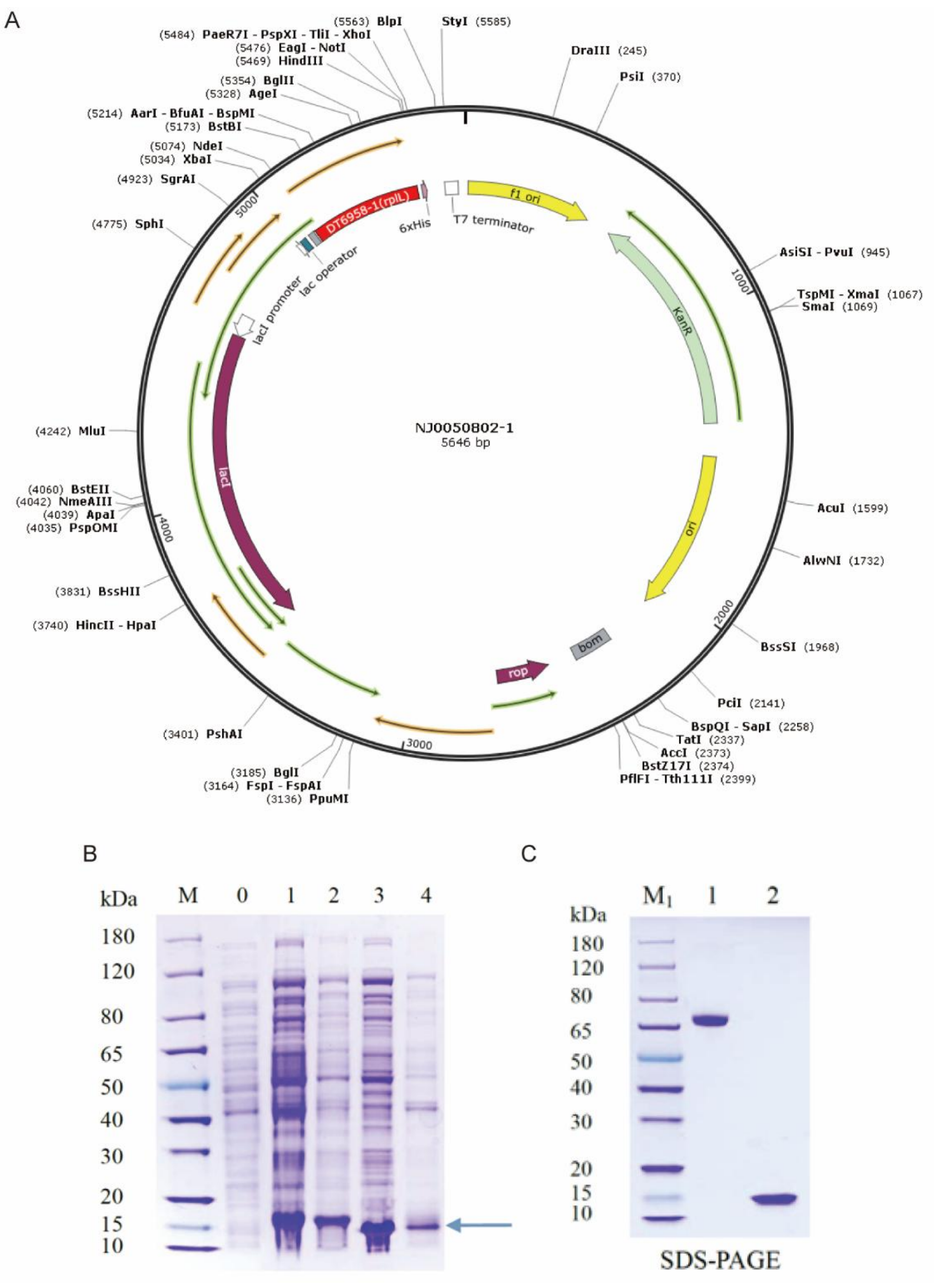

Figure S1. Recombinant expression plasmid and SDS-PAGE analysis of ribosomal L7/L12 protein. (A) Schematic representation of the pET30a expression vector harboring the *RplL* gene. (B) SDS-PAGE analysis of RplL expression in BL21(DE3). Lane M: protein marker; Lane 0: uninduced control (without IPTG); Lane 1: induction at 15 °C for 16 h; Lane 2: induction at 37 °C for 16 h; Lane 3: supernatant after cell lysis; Lane 4: pellet after cell lysis. (C) SDS-PAGE analysis of purified RplL protein. M1: SDS-PAGE protein marker; Lane 1:

BSA standard (1 µg); Lane 2: purified RplL protein (1.2 µg).

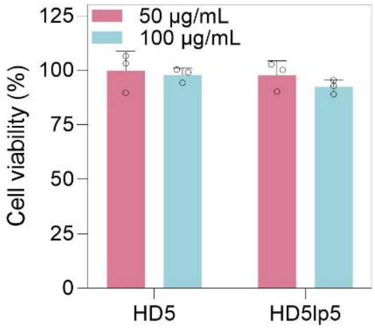

Figure S2. Survival of HUVEC cells exposed to peptides after 24 h. The results are shown as the mean ± SD of three biological replicates (n = 3), with technical replicates averaged prior to analysis.

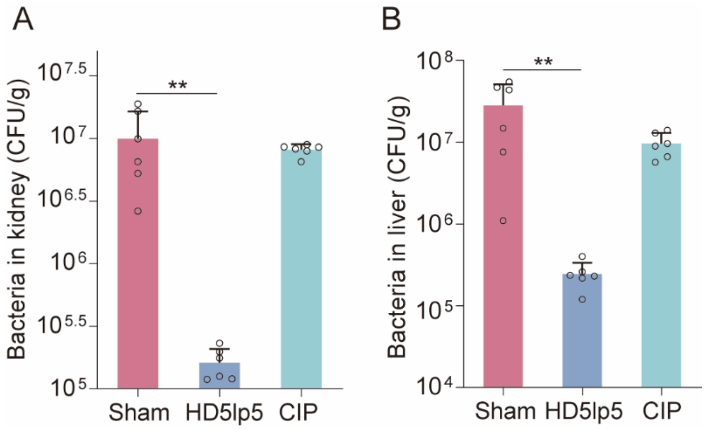

Figure S3. Bacterial counts in mice kidney (A) and liver (B). The results are shown as the mean ± SD of six biological replicates (n = 6). **, $p < 0.01$, as determined by one-way ANOVA followed by Tukey's multiple comparisons test.

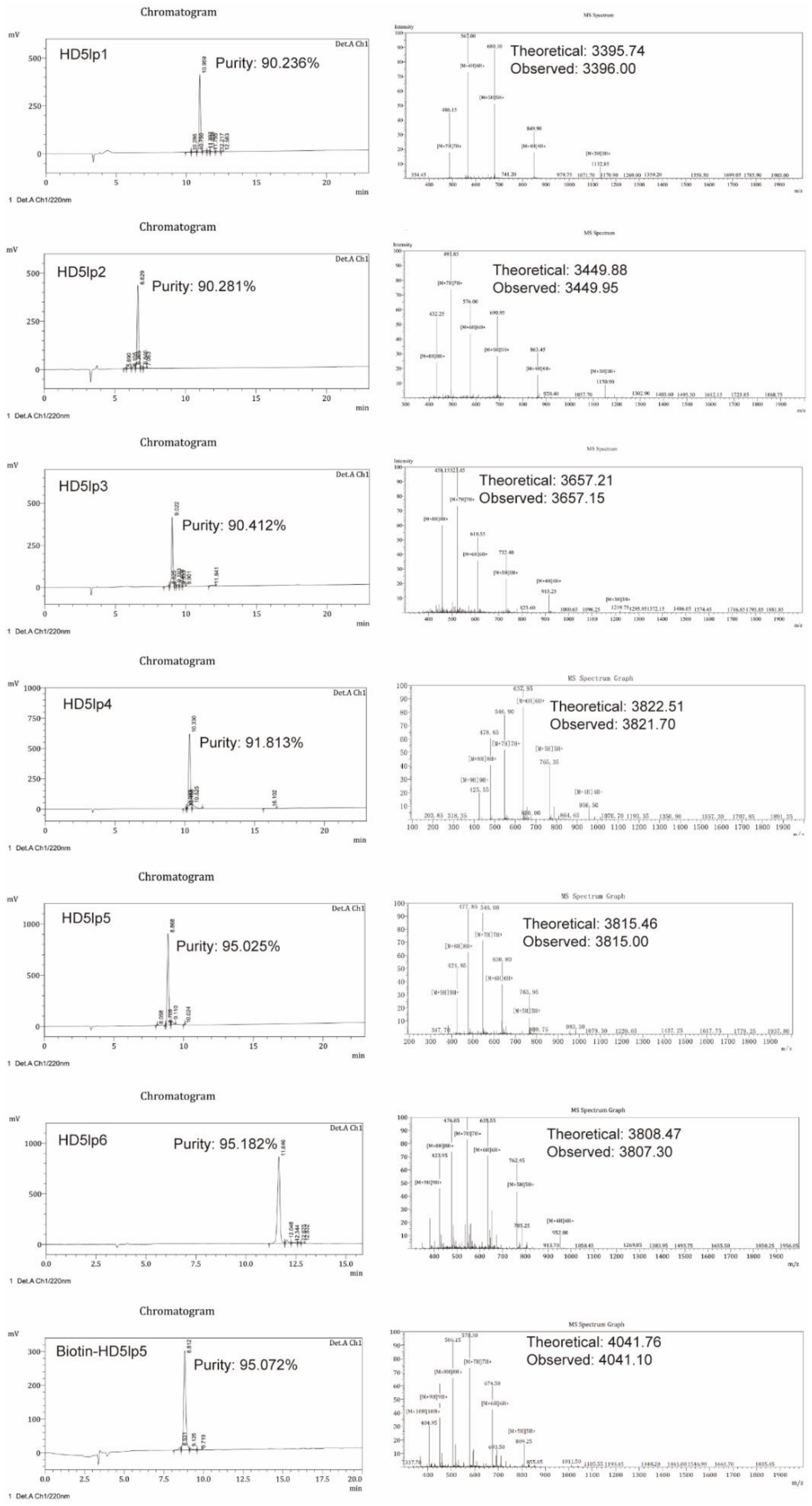

Figure S4. Detailed purities and MWs of synthetic peptides. Peptide purities are all above 90%. The observed MWs of these peptides are close to their theoretical values.
